# Self-DNA driven inflammation in COVID-19 and after mRNA-based vaccination: lessons for non-COVID-19 pathologies

**DOI:** 10.3389/fimmu.2023.1259879

**Published:** 2024-02-19

**Authors:** Martin Heil

**Affiliations:** Departamento de Ingeniería Genética, Laboratorio de Ecología de Plantas, Centro de Investigación y de Estudios Avanzados (CINVESTAV)-Unidad Irapuato, Irapuato, Mexico

**Keywords:** self-DNA, DAMPs, HIV-1, immunogenic DNA, mRNA vaccines, COVID-19, autoimmune disorder, sterile inflammation

## Abstract

The coronavirus disease 2019 (COVID-19) pandemic triggered an unprecedented concentration of economic and research efforts to generate knowledge at unequalled speed on deregulated interferon type I signalling and nuclear factor kappa light chain enhancer in B-cells (NF-κB)-driven interleukin (IL)-1β, IL-6, IL-18 secretion causing cytokine storms. The translation of the knowledge on how the resulting systemic inflammation can lead to life-threatening complications into novel treatments and vaccine technologies is underway. Nevertheless, previously existing knowledge on the role of cytoplasmatic or circulating self-DNA as a pro-inflammatory damage-associated molecular pattern (DAMP) was largely ignored. Pathologies reported ‘*de novo*’ for patients infected with Severe Acute Respiratory Syndrome Coronavirus (SARS-CoV)-2 to be outcomes of self-DNA-driven inflammation in fact had been linked earlier to self-DNA in different contexts, e.g., the infection with Human Immunodeficiency Virus (HIV)-1, sterile inflammation, and autoimmune diseases. I highlight particularly how synergies with other DAMPs can render immunogenic properties to normally non-immunogenic extracellular self-DNA, and I discuss the shared features of the gp41 unit of the HIV-1 envelope protein and the SARS-CoV 2 Spike protein that enable HIV-1 and SARS-CoV-2 to interact with cell or nuclear membranes, trigger syncytia formation, inflict damage to their host’s DNA, and trigger inflammation – likely for their own benefit. These similarities motivate speculations that similar mechanisms to those driven by gp41 can explain how inflammatory self-DNA contributes to some of most frequent adverse events after vaccination with the BNT162b2 mRNA (Pfizer/BioNTech) or the mRNA-1273 (Moderna) vaccine, i.e., myocarditis, herpes zoster, rheumatoid arthritis, autoimmune nephritis or hepatitis, new-onset systemic lupus erythematosus, and flare-ups of psoriasis or lupus. The hope is to motivate a wider application of the lessons learned from the experiences with COVID-19 and the new mRNA vaccines to combat future non-COVID-19 diseases.

## 1 Introduction

“*Nucleic acids are one of the few molecular patterns that can be used to detect viruses*”. Jacques Deguine, 2017 (1)

Coronavirus disease 2019 (COVID-19) caused by infection with Severe Acute Respiratory Syndrome Coronavirus (SARS-CoV)-2 was initially considered as an infectious inflammatory lung disease. Yet, it became clear quite quickly that severe cases of COVID-19 comprise systemic endothelial dysfunction and inflammation in respiratory and non-respiratory organs that result from deregulated type I interferon (IFN I) signalling (2–7). During the initial stage of infection, a rapid production of IFN I and of IFN-stimulated genes, including several inflammatory cytokines and chemokines (the so-called IFN I response), can protect surrounding cells from infection and thus, usually is sufficient to halt viral replication (8, 9). However, during later stages of the infection cycle, SARS-CoV-2 triggers an ongoing expression and/or activation of the transcription factor nuclear factor kappa light chain enhancer in B-cells (NF-κB) and downstream, of tumour necrosis factor (TNF)-α, interleukin (IL)-1β, IL-6, IL-18 (hereinafter termed ‘pro-inflammatory cytokines’) and IFN-γ (the only type II IFN), with little contribution of antiviral IFN I/III (3, 10). The resulting cytokine storm (11) sustains detrimental inflammation and drives massive bystander cell death, thereby generating endothelial damage in multiple organs and – most likely – causing the progress to severe forms of COVID-19, with potentially fatal outcomes (3, 7, 10, 12–16).

**Figure 1:**
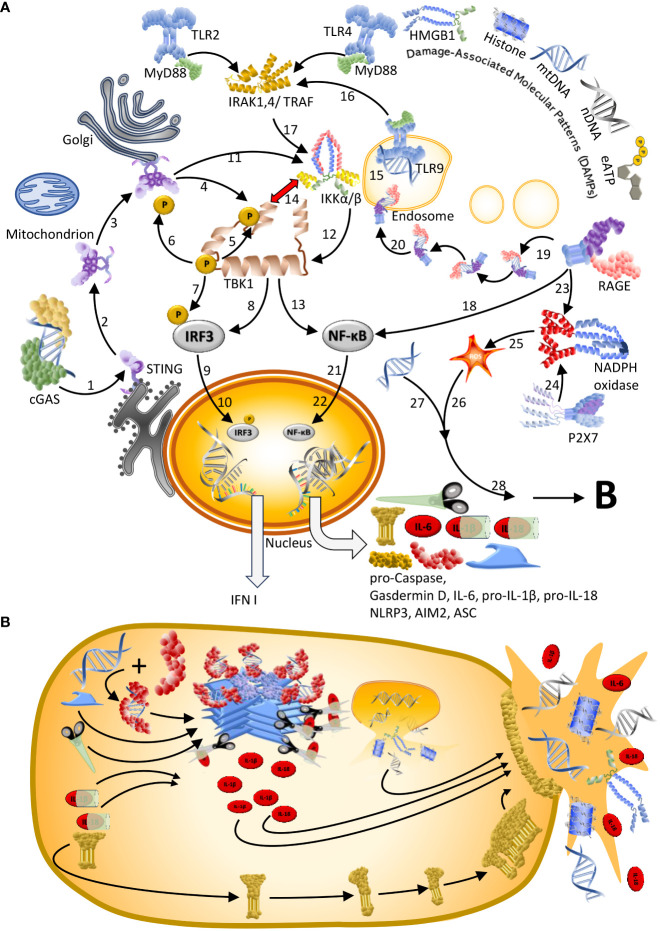
Sensors of dsDNA and downstream signalling pathways. **(A)** Double-stranded (ds)DNA sensors and signalling cascades reported in the context of COVID-19. [**1**] Upon dsDNA binding, cyclic GMP-AMP synthase (cGAS) produces cyclic GMP-AMP (cGAMP) to activate stimulator of interferon genes (STING): an adaptor protein that in homeostasis resides as monomer in the endoplasmic reticulum. [**2**] Upon activation by cGAMP, STING oligomerises and [**3**] translocates to the Gologi Apparatus to recruit [**4**] TANK binding kinase 1 (TBK1). [**5–7**] Subsequently TBK1 phosphorylates itself, STING, and interferon regulatory factor 3 (IRF3) (26), thereby [**8**] activating IRF3. [**9**] Activated IRF3 moves into the nucleus to [**10**] trigger the transcription of type I and type III interferon (IFN I). In patients with COVID-19, cGAS/STING have been described to trigger a distinct response and activate nuclear factor kappa light chain enhancer in B-cells (NF-κB), either [**11**] directly via the IκB kinase (IKK) complex (27), [**13**] which then activates NF-κB, or [**13**] indirectly via a TBK1-dependent activation and [**14**] a mutual inhibition between TBK1 and IKK (27) along with a block of IRF3 nuclear translocation (15). More common activators of NF-κB are Toll-like receptors (TLRs), including the endosome-expressed TLR9, which is the only known sequence-specific DNA sensor. [**15**] Upon activation by endosomal CpG-rich dsDNA, TLR9 – like most TLRs – associates with the adaptor protein myeloid differentiation primary response (MyD)88 to [**16**] facilitate the formation of a complex with Interferon receptor-associated kinases (IRAKs) and TNF receptor-associated factor (TRAF)6. [**17**, **12**] Downstream, TAK1 and IKKs activate NF-κB. [**18**] A further receptor that signals via NF-κB is the receptor for advanced glycation end products (RAGE), the only dsDNA sensor in the cell membrane. [**2**19] Furthermore, RAGE can internalize with its ligand and [**20**] deliver it to the endosome to facilitate sensing by TLR9, which ultimately activates NF-κB and – likely – amplifies the inflammatory response. [**21**] Activated NF-κB translocates to the nucleus to [**22**] facilitate the expression of pro-IL-1β, IL-6, pro-IL-18, and of the different elements of the inflammasome: AIM2, NLRP3, apoptosis-associated speck containing a caspase recruitment domain (CARD) (ASC)-like protein, pro-caspase and pro-gasdermin D. Thereby, agonists of TLRs prime the cell for inflammasome formation. Activation of the inflammasome requires a second signal. [**23**] RAGE can provide this signal by activating NADPH oxidase. [**24**] Alternatively, NADPH oxidase can be activated by the P2X7 receptor in response to sending extracellular ATP (eATP). [**25**] The reactive oxygen species (ROS) formed by NADPH oxidase can function as signal II [**26**] and activate the inflammasome. [**27**] Alternatively, cytoplasmatic DNA that is sensed by AIM2 or NLRP3 can act as signal II. (**B**) Upon sensing signal II, AIM2 or NLRP3 associate with ASC and pro-caspase 1 to form the active inflammasome that liberates active caspase-1, thereby facilitating the maturation of IL-1β and IL-18. Since these ILs don´t possess a secretion signal, active gasdermin D is also produced to form a plasma membrane pore, which enables the release of these cytokines together with cellular content. See **Figure 2** for graphical legend.

Several studies identified DNA-sensing pattern recognition receptors (PRRs) as drivers of IFN I-driven inflammation and massive cell death in severe COVID-19: cyclic GMP–AMP synthase (cGAS), absent in melanoma (AIM)2, nucleotide-binding oligomerization domain (NOD)-like leucine-rich repeat (LRR) and PYRIN domain containing (NLRP)3, receptor for advanced glycation end products (RAGE), and Toll-like receptor (TLR)9 (9, 13, 14, 17–23): Upon detecting double stranded (ds)DNA, these PRRs activate the production of antiviral and pro-inflammatory cytokines and chemokines and eventually, cell death, via two principal pathways (reviewed in (24, 25)). While cGAS signals via stimulator of interferon genes (STING) to induce predominantly IFN I, TLRs signal via the adaptor protein myeloid differentiation primary response (MyD)88 and the transcription factor NF-κB to activate the expression of IL-1β, IL-6 and IL-18 and of AIM2, NLRP3 and other elements of the inflammasome. Inflammasomes are multiprotein complexes that control the massive release of pro-inflammatory cytokines via pyroptosis, a pro-inflammatory cell death (**Figure 1**, see refs (38–40) for reviews).

**Figure 2:**
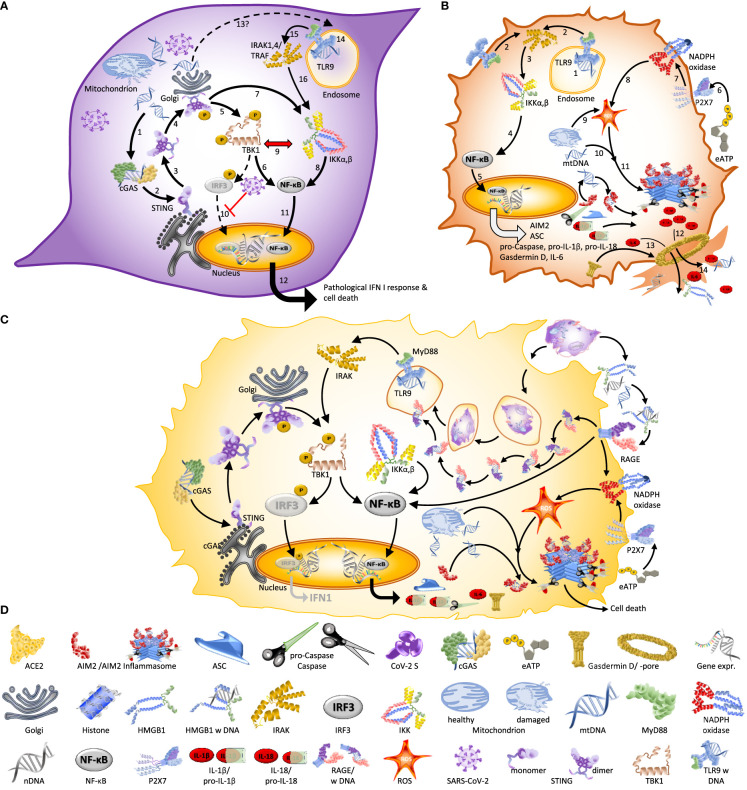
Different forms of cell death associated with SARS-CoV-2 infection are driven by DNA. **(A)** Autonomous cell death of infected endothelial cells and epithelial driven by cGAS or TLR9 (9, 15, 18). Infection with SARS-CoV-2 damages mitochondria and leads to the release of mtDNA to the cytosol. [**1–4**] This mtDNA activates cGAS/STING signalling, which [**5–6**] activates NF-κB via TBK1 or [**7–8**] IKKs. A dominance of NF-κB over IRF3-dependent signalling is favoured by [**9**] the mutual inhibition of TBK1 and IKKs and [**10**] SARS-CoV-2 blocking the translocation of IRF3 to the nucleus. [**11**] In consequence, NF-κB moves into the nucleus to [**12**] trigger expression of NF-κB-dependent genes; an effect which - if sustained during the late phase of the infection - leads to aberrant inflammation and poor clinical outcome (9). [**13**–**16**] Others reported mtDNA to become - via an unknown mechanism – accessible to TLR9 and thereby trigger IRAK1/4 and TRAF6-dependent activation of IKKs. **(B)** Inflammasome-driven cell death of infected monocytes and macrophages (22, 31, 32) typically requires a first, priming stage in which [**1**] a TLR ligand such as, e.g., endosomal dsDNA, triggers the association of a TLR with the adapter protein MyD88 to [**2**–**3**] activate IKK via IRAK/14, TRAF6 and other downstream protein complexes. [**4**] Subsequently activated NF-κB and [**5**] NF-κB-driven expression of the inflammasome components absent in melanoma2 (AIM2), (NOD)-like leucine-rich repeat (LRR) and PYRIN domain containing3 (NLRP3), ASC-like protein, and the precursors of caspase 1, Gasdermin D, Il-1β and IL-18 allow the cell to enter the primed stage. The subsequent activation of the inflammasome can be initiated by diverse cytoplasmatic DAMPs, but also, e.g., by extracellular ATP (eATP). [**6**] eATP is sensed by P2X7, [**7**] which subsequently activates membrane-bound NADPH oxidase to [**8**] generate intracellular reactive oxygen species (ROS). Alternatively, [**9**] mitochondrion-derived cytoplasmatic ROS or [**10**] mtDNA have been suggested to act as signal II (18) that [**11**] triggers the assembly of the inflammasome (as illustrated here for the AIM2 inflammasome). [**12**] Inflammasome-liberated Il-1β and IL-18 [**13**] and IL-6 [**14**] are released via the membrane pore formed by Gasdermin D together with other cellular content, a process culminating in pyroptosis. **(C)** Bystander cell death of macrophages. Self-DNA from dying infected endothelial cells that have been engulfed by macrophages can activate TLR9 in these cells to trigger NF-κB-controlled inflammatory signalling. This pathway can be amplified when extracellular DAMPs like mtDNA or nDNA are sensed by RAGE, which then directly activates NF-κB, or by a receptor-assisted transport of DNA or DNA-protein complexes to the endosome, where these can be detected by TLR9 (33). It is likely that this mechanism explains the association of plasma levels of RAGE in the blood of COVID-19 patients with poor clinical outcome (34, 35). **(D)** Graphical legend of **Figures 1**–**3**.

At first glance, the identification of dsDNA-sensors as players in coronavirus disease seems difficult to understand. SARS-CoV-2 is a single stranded (ss)RNA virus and, unlike retroviruses such as HIV-1, coronaviruses do not reverse-transcribe their RNA genome to DNA. So, if no viral cDNA is synthesized, which DNA activates innate immunity in COVID-19? It turns out that fragments of the host’s ‘self-DNA’ activate the before mentioned dsDNA sensors to trigger - eventually detrimental - inflammation and cell death (**Figure 2**). Several groups reported that SARS-CoV-2 infection generates oxidative stress, damages the mitochondrial genome, destabilizes the mitochondrial membrane and subsequently, triggers a release of mitochondrial (mt)DNA to the cytosol (9, 13, 18, 41, 42). Thereby, mtDNA becomes accessible to cGAS, AIM2 or NLRP3 (9, 18, 22). Second, SARS-CoV-2-infected cells can undergo syncytia formation, a cell-to-cell fusion that generates multi-nucleated cells and therefore, is associated with DNA damage, nuclear membrane blebbing and a release of chromatin – including nuclear (genomic) DNA (nDNA) - to the cytosol, where it is sensed by cGAS (9, 13, 17, 23, 36). Moreover, the DNA of dying infected cells can trigger inflammation and pyroptotic cell death in immune bystanders, either because the DNA of engulfed cells becomes exposed to TLR9, or because DNA that these cells release to the extracellular space serves as an inflammasome-activating signal (9, 13).

Elevated concentrations of cell-free (cf) self-DNA in blood or plasma of COVID-19 patients have been reported repeatedly and in most cases, cfDNA levels correlated with disease severity (13, 18–21, 42–56). This finding should not come as a surprise, because during pyroptosis, pro-inflammatory cytokines are released together with cellular content, including self-DNA and other damage-associated molecular patterns (DAMPs) (6, 13, 14, 46, 54, 57, 58). DAMPs (or alarmins) are endogenous molecules that adopt the additional function of signalling danger when their fragmentation or translocation to the ‘wrong space’ indicates damage to self (59–61). Sensing self-DNA as a DAMP allows the detection of harmful agents directly based on the harm they do (62). For example, the rapid pyroptotic cell death of SARS-CoV-2-infected blood monocytes or lung-resident macrophages prevents the virus from completing its reproductive cycle (22, 28).

However, DAMPs-mediated signalling can also generate detrimental effects, particularly when a massive release of pro-inflammatory DAMPs from dying cells causes more cells to die: a situation that strongly contributes to the CD4+ T cell depletion in patients infected with HIV-1 (63, 64), reviewed in (65). Extracellular self-DNA has been associated with multiorgan failure after severe trauma (66–70), including sepsis-like systemic inflammation (71), sepsis (72), can lead to myocarditis (73–76) or lung inflammation (77), facilitates the reactivation of latent virus infections, including Herpes Simplex Virus, Varicella Zoster Virus, or HIV-1, and favours the development of autoimmune diseases such as systemic lupus erythematosus (SLE) and psoriasis (67, 76, 78–86). Elevated plasma levels of cf nDNA or mtDNA are common in HIV-1-infected patients (87–91) and might explain the chronic inflammatory and autoimmune-related pathologies that frequently develop in this group (65, 92–94): examples include thrombocytopenia (95–98), diverse forms of vasculitis (99, 100), myocarditis (101), psoriasis (102–104), rheumatoid arthritis (105, 106), SLE (102, 105, 106) and herpes zoster (107).

Considering the role of circulating DNA in these pathologies and the similarity of some of the resulting symptoms, a role for circulating self-DNA in severe COVID-19 appears highly likely. Nevertheless, it seems that the potential importance of pro-inflammatory self-DNA had to be discovered ‘*de novo*’ for COVID-19. The first studies that associated increased serum levels of certain DAMPs with severe COVID-19 appeared in 2020 (108–111). Correspondingly, a role of immunogenic self-DNA in severe COVID-19 was hypothesised in the same year (112) and indeed, a team from the University of Missouri with first-author Alex Earhart were the first to publish on 30th of April 2020 empirical evidence for a role of extracellular DNA in severe COVID-19: the authors used a DNase (Dornase α) to dissolve DNA-containing neutrophil extracellular NETs - large, extracellular webs formed by cytosolic proteins and decondensed chromatin including histone and n/mtDNA that trap bacteria or viruses (113–115) - in the lung of a COVID-19 patient (116), Subsequently, others followed the rationale that the degradation of extracellular DNA by Dornase α should reduce mucus rigidity and accumulation and thereby lead to respiratory improvement (117–122). Importantly, a group from S. Korea with first author Hee Ho Park (119) reported on Oct 20th cfDNA concentrations of 0.41 mg mL^-1^ in the blood of healthy individuals and of 0.85 and 2.83 mg mL^-1^ in the blood of patients with light and severe COVID-19, respectively (119).

With exception of the before mentioned studies, however, there is little evidence that existing knowledge on inflammatory self-DNA guided the research towards mechanisms that lead to severe COVID-19 or the trials aimed at repurposing pre-existing drugs during the first phase of the COVID-19 pandemic. Even less evidence indicates that knowledge on how and why certain viruses trigger DNA damage had any influence on vaccine development. Although one can only speculate about the reasons, it seems possible that papers published in 2003 or 2004 were rated outdated, at least by the younger generation of scientists, while HIV-1 was possibly deemed ‘too distant’. In addition, it appears that the immunogenic properties of self-DNA and its potential roles in multiple pathologies has not yet been fully assimilated by the immunological community, perhaps because self-DNA as a paramount DAMP contradicts the immunological paradigm of self-tolerance. As pointed out by the legendary immunologist Andrea Ablasser, “such a ‘universal’ sensing mechanism violates one of the most fundamental rules of the classical pattern recognition dogma, which is based on pathogen-specific structural patterns instructing self- versus nonself discrimination” (123).

Therefore, the aim of the present work is to motivate a wider application of the lessons learned from the experiences with COVID-19 and the new mRNA vaccines to combat future non-COVID-19 diseases, providing preliminary and associational evidence for the hypothesis: "Self-DNA-driven inflammation is one of the factors that contribute to severe COVID-19 and to certain adverse events subsequent to COVID-19 mRNA vaccination.

This hypothesis has mainly been formulated considering that:

1. Immunogenic self-DNA has been proposed as a driver of inflammatory and autoimmune-related processes in HIV-1-infected patients.
2. Similar, if not identical, symptoms have been observed among HIV-1-infected and SARS-CoV-2-infected patients as well as in vaccinated individuals who suffer from severe adverse effects (12).
3. Elevated levels of cf nDNA or mtDNA have been detected in plasma of HIV-1-infected patients (87–91) and of SARS-CoV-2-infected patients (19, 20, 42–44, 47, 49, 52–56, 119), as well as in the supernatant of SARS-CoV—infected human airway epithelial cells (13, 18).
4. Elevated levels of autoantibodies, in particular anti-dsDNA and antinuclear antibodies, have been reported from patients with severe COVID-19 in various case reports, but also in a study that compared 217 COVID-19 patients in the ICU with 117 age- and sex-matched controls (124) (for a review see (125)).
5. Multiple reports on elevated levels of anti-dsDNA antibodies in the plasma of vaccinees (126–132) indicate that increased cfDNA levels might be common in this group, although direct evidence for elevated levels of cfDNA in plasma of vaccinated individuals is scarce (but see (127)).
6. A mouse model demonstrated that CoV-2 S expression caused enhanced levels of autoantibodies and inflammatory cytokines, which ultimately led to tissue destruction (133), and
7. a long-lasting persistence of full-length CoV-2 S protein has been reported from plasma of several individuals who presented post-vaccination myocarditis, but not from vaccinated individuals who did not suffer from adverse effects (134).

To support this hypothesis, rather than providing a balanced review, I discuss mechanisms by which self-DNA has been reported – or suggested – to drive inflammation with potentially detrimental effects in non-COVID-19 pathologies and which are highly likely to apply also in COVID-19. I focus particularly on HIV-1, because SARS-CoV-2 and HIV-1 share multiple features, among others in the functions of CoV-2 S and the gp41 unit of the envelope glycoprotein of HIV-1 (135). For parallels with inflammaging see (136–139) and those with autoimmune diseases see (86).

## 2 Evidence from other pathologies

The role of various DAMPs in COVID-19 has been reviewed by Søren Paludan and Trine Mogensen (140). Self-DNA, in particular, has received less attention, although existing knowledge from several non-COVID pathologies provides a solid basis to hypothesize a similar role in COVID-19. Over decades, self-DNA was considered as immunologically inert, although the cytokine-activity of DNA was known many years before its identification as the carrier of the genetic information (141). During homeostasis, diverse cytosolic and extracellular DNases eliminate self-DNA that appears outside the nucleus and in addition, the cytoplasmatic expression of dsDNA sensors was believed sufficient to avoid erroneous immune responses to self-DNA: dying cells will usually release their DNA into the extracellular space, and DNA – being a heavily negatively charged molecule – does not normally pass through membranes. However, in complexes with positively charged molecules, including certain DAMPs, DNA can become immunogenic (61, 142).

As mentioned in the introduction, self-DNA has been associated with multiorgan failure after severe trauma, myocarditis, the reactivation of latent virus infections, including Herpes Simplex Virus, Varicella Zoster Virus, or HIV-1, thrombocytopenia, psoriasis (102–104), rheumatoid arthritis and SLE. Intriguingly, these pathologies were also common among COVID-19 patients (39, 137, 143), and the transcriptome of skin lesions from patients with severe COVID-19 exhibited strong similarities with cutaneous LE (9). Similar symptoms emerged among the most frequent severe adverse events subsequent to COVID-19 vaccination, at least for some of the platforms (144). For example, thrombocytopenia was mainly observed among vaccinees who received the ChAdOx1 adenoviral-vector-DNA (AstraZeneca) vaccine (145, 146) and cutaneous vasculitis was observed mainly in those receiving ChAdOx1 (147) or the BNT162b2 mRNA (Pfizer-BioNTech) vaccine (148, 149) (reviewed in (150)). Most of the other disorders identified as severe adverse effects were reported predominantly from individuals who received the BNT162b2 vaccine, followed by mRNA-1273 (Moderna): examples include myocarditis (151–157), herpes zoster (154, 158–160), rheumatoid arthritis (161, 162), autoimmune nephritis or hepatitis (132, 163), new-onset SLE (126, 128, 130) or neurological autoimmunity (164), and flare-ups of psoriasis (154, 165–168), or SLE (129, 152, 161, 162, 169).

All these pathologies could theoretically be driven – partly or completely – by immunogenic self-DNA and in fact, all of them have been related to self-DNA in the context of non-COVID-19 pathologies. Using trauma as an example, several prospective observational studies reported elevated concentrations of free cfDNA (66–68, 70), or of histone-complexed cfDNA (170), in the blood of severely injured trauma patients. In all these studies, the cfDNA concentrations correlated with poor clinical outcome (66–68, 70, 170). The causal role cfDNA in the inflammatory response to trauma was shown by the group of Carl Hauser at Harvard (67, 69). Back in 2010, the group observed increased plasma levels of mtDNA and nDNA in rats exposed to traumatic injury combined with haemorrhagic shock and found that hepatocyte-derived mtDNA, but not nDNA, activated polymorphonuclear neutrophils *in vitro* (69). The group compared the activation of various mitogen-associated protein kinases (MAPKs) and used endosome acidification assays to identify TLR9 as the most likely receptor of this mtDNA. *In vivo*, mtDNA delivered via tail-vein injection triggered liver inflammation at 1hr that was associated with enhanced levels of IL-6 and TNF-α in whole liber homogenate (69). More recently, the group demonstrated that treatment with a nucleic acid scavenger (polymerous hexadimethrine bromide) can rescue the rodents from severe multiple organ dysfunction (67).

## 3 Self-DNA as a DAMP in COVID-19

“*Coming across extracellular DNA and RNA swimming around when you are a cell is usually bad news*”. Sophia Häfner, 2013 (171)

### 3.1 Signalling pathways triggered by dsDNA

As mentioned in the introduction, several PRRs sense DNA (see **Figure 1**) and, with exception of TLR9, they do so in a sequence-independent manner. This means that in principle, these PRRs do not distinguish self- from nonself-DNA. This lack of a specificity for any sequence motifs explains why cGAS is now considered one of the elements that connect DNA damage to several autoimmune diseases and cancer, but also the counterintuitive observation of cGAS-controlled immunity against RNA viruses, including HIV-1, human T cell-leukaemia virus type I and Dengue virus (172–174). Active cGAS produces cyclic GMP-AMP to activate STING, which dimerises and activates TNF receptor associated factor (TRAF) associated NF-κB activator (TANK) binding kinase 1 (TBK1) and downstream, interferon regulatory factor 3 (IRF3), which moves into the nucleus and triggers the transcription of type I and type III interferon (IFN). In the particular case of SARS-CoV-2, the cell-autonomous activation of cGAS/STING signalling also contributes to NF-κB-dependent cytokine production, at least in human epithelial cells (15).

Besides cytosolic cGAS, endosomal TLR9 has been reported to be activated by SARS-CoV-2 (18, 175). TLRs are membrane-bound PRRs that sense a diverse array of extracellular or endosomal DAMPs and PAMPs and form part of one of the most studied examples of a DAMP-mediated immune priming: the activation of inflammasomes in two steps (59, 176, 177). Upon binding by one of these ligands, most TLRs signal via myeloid differentiation primary response (MyD)88, Interferon Receptor-Associated Kinases (IRAKs), TNF receptor-associated factor (TRAF)6, TBK1, and the IKK complex (69, 178). Downstream, activated NF-κB translocates to the nucleus to facilitate the expression of pro-IL-1β, IL-6 and pro-IL-18 and of the different elements of the inflammasome: AIM2, NLRP3, apoptosis-associated speck containing a caspase recruitment domain (CARD) (ASC)-like protein, pro-caspase, and pro-Gasdermin D (**Figure 1A**) (40, 58, 179). Thereby, TLR ligands serve as signal 1 that primes the cell for fast responses to future, more challenging threats, which are indicated by a second, intracellular signal 2. Both AIM2 and NLRP3 are dsDNA sensors that bind to cytoplasmatic DNA, including phagocytosed DNA that is released from lysosomes. AIM2 seems to exclusively sense DNA, but NLRP3 senses multiple endogenous and exogenous molecules that indicate threats, including reactive oxygen species (ROS), and it is also activated when extracellular ATP (eATP) binds to a purinergic receptor, e.g. P2X7 (38, 59, 180). In response to this ‘activation’ signal, AIM2 or NLRP3 associate with ASC and pro-caspase 1 to form the active inflammasome, a multiprotein complex that liberates active caspase-1 to facilitate the maturation of IL-1β and IL-18. Since these ILs don´t possess a secretion signal, active gasdermin D is also produced to form a plasma membrane pore, which enables the release of these cytokines together with cellular content (13, 181) (**Figure 1B**).

### 3.2 Pathways to DNA-driven inflammatory cell death in COVID-19

In the context of COVID-19, the dsDNA sensors participate in inflammatory processes that usually culminate in cell death, via mechanisms that can be roughly grouped into three major pathways: Cell-autonomous cell death, bystander cell death and dying syncytia. Cell-autonomous cell death leading to aberrant inflammation in COVID-19 (**Figure 2A**) has been described, e.g., by Andrea Ablasser’s team in Lausanne (9) and Ralf Bartenschlager’s team in Heidelberg (15) as an outcome of mtDNA release from damaged mitochondria that results in an ‘aberrant’ cGAS/STING-pathway causing a specific activation of NF-κB and a block of IRF3 nuclear translocation in SARS-CoV-2 infected endothelial cells and lung epithelial cells, and by a Brazilian team with first author Tiago Costa (18) as the outcome of a mitochondrial dysfunction that leads to a TLR9-dependent NF-κB activation in human umbilical vein endothelial cells (**Figure 2A**). Furthermore, a team at NIH in Bethesda and Georgetown University in Washington (29) described autonomous cell death to be caused NLRP3-inflammasomes activation by high levels of oxidative stress that are associated with mitochondrial dysfunction in human monocytes (29), and also Judy Lieberman’s and Richard Flavell’s teams at Harvard Medical School and Yale (22, 28) inflammasome-dependent pyroptosis of SARS-CoV-2 infected macrophages and monocytes (**Figure 2B**). Doubts remain, and several differences among these papers will have to be resolved by future work. For example, Junqueira et al. observed ca.10 % infected cells among the blood monocytes in COVID-19 patients (22), while Lage et al. “were unable to detect productive infection of primary human HC monocytes by SARS-CoV-2 *in vitro*” (29). Di Domizio et al. (9) define endothelial cell death as STING-dependent but do not offer a concrete mechanism that connects aberrant IFN I-signalling to the death of infected cells and – curiously enough – I could not find a single study that considered both cGAS/STING signalling and inflammasomes together. Doubts also remain concerning the signals I and II that are generated by SARS-CoV-2 replication to activate inflammasomes in the same cell and whether activation of TLR9 was caused by mtDNA or a direct effect of the virus. Both mitochondrial ROS (29) and DNA from other dying cells (Lieberman, personal communication) were suggested to activate inflammasomes as signal II, which in the latter case would classify the cell death as bystander cell death.

Evidently, infected cells eventually die. Before or after doing so, they can trigger inflammation and eventually, cell death, in neighbouring immune ‘bystander’ cells. The team headed by Andrea Ablasser (9) observed that activated, IFN I-producing macrophages frequently surrounded those vessels that exhibited strongest signals of endotheliopathy and concluded that “signals derived from dying (endothelial) cells promote type I IFN production by macrophages”. Similarly, a particularly nice and detailed study performed in Jenny Ting’s Lab in Chapel Hill and published by Katherine Barnett as first author (13) started from immunohistochemical analyses of COVID-19 autopsy lungs, which showed active inflammasomes and cell death in alveolar macrophages directly adjacent to infected alveolar epithelial cells. Subsequently, they used a co-culture system of human airway epithelial cells and peripheral blood mononuclear cells to confirm that inflammasome activation and cell death was limited to co-cultured cells but absent in isolated cells. Both studies suggested self-DNA (both mitochondrial and genomic) from the dying infected cells as the signal that activated inflammation in the immune cells, although Di’Domizio et al. favoured engulfed endothelial cells (**Figure 2C**) while Barnett et al. suggest lytic cell death as the mechanism allowing bystander cells to access DNA from infected cells (9, 13).

**Figure 3:**
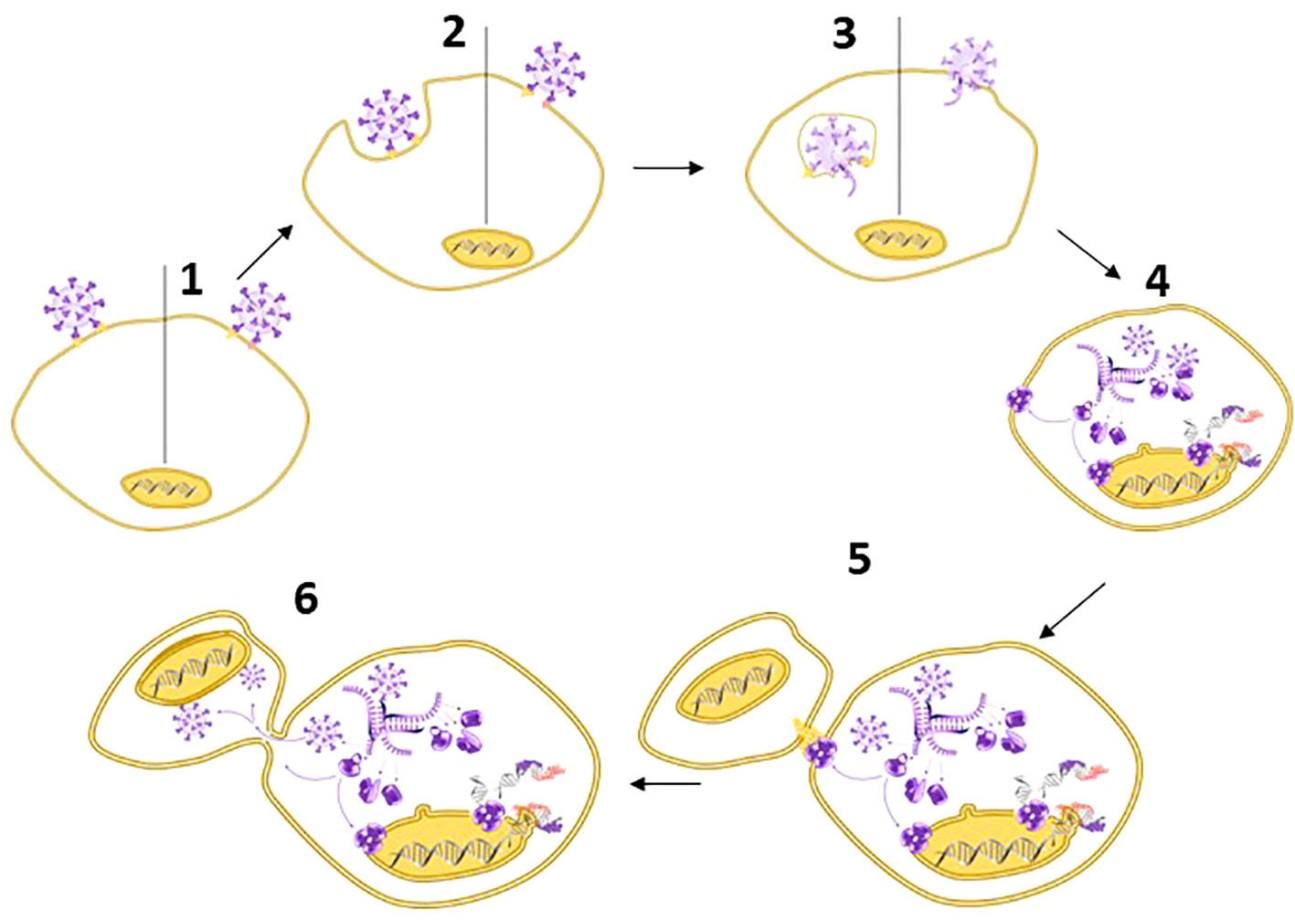
Spike – the multipurpose locksmith. Common knowledge holds that the spike protein serves to recognize specific receptors on the surface of host cells, but in fact, CoV-2 S has several roles: Binding to a cell membrane receptor, mediating membrane fusion between the viral and host membranes, which results in the release of viral content, syncytia formation, and – likely – the destabilisation of the nuclear envelope (33–35). [1] Viral entry can occur via endocytosis or membrane fusion. Both processes depend on CoV-2 S, [2] independently of whether entry occurs via the endosomal pathway (left) or membrane fusion (right). After entry [3], viral RNA is released into the cytoplasm and [4] used for the replication of the RNA genome and protein synthesis. This process is associated with DNA damage, a destabilization of the nuclear envelope, and nuclear membrane blebbing (→): processes that likely benefit the virus because they liberate the nucleic acids that SARS-CoV-2 requires for its replication. [5-6] Subsequently, Spike protein expressed on the cell surface (→) also controls syncytia formation: a process that usually serves to facilitate viral spread without exposure to the host immune system (34, 36). The first virus for which nuclear membrane blebbing has been reported is HIV-1 (37). In the case of HIV-I, the bipartite envelope glycoprotein (Ev)fusion performs the two essential functions of binding to receptors on the surface of target cells and fusioning the host-cell and viral membranes, including the formation of a fusion pore to deliver the viral core into the cell cytoplasm.

Third, SARS-CoV-2-infected cells can fuse with non-infected ACE2-expressing cells to form syncytia: multinucleated cell complexes that facilitate viral spread without exposure to host antibodies (23, 182, 183) (**Figure 3**). Various groups observed syncytia as a common cytological feature of post-mortem lung samples obtained from individuals who died of COVID-19 (23, 182, 184–186). A team from Beijing and Harvard with first author Zhou Zhuo (17) focused on the switch from a suppression of type I IFN signalling during the initial phase of infection to the (over-) induced cytokine signalling at later stages and discovered that this shift is associated with syncytia formation, which in turn is accompanied by the release of chromatin – including nDNA to the cytosol (13, 17). Thereby, SARS-CoV 2 enhances the visibility of its’ hosts self-DNA to the immune system (187).

### 3.3 Self-DNA as a DAMP in COVID-19: preliminary evidence

Pharmacological attempts to support a causal role of DNA in COVID-19 used DNA-scavenging nanomaterials or recombinant DNase-I (Dornase α). Bruce Sullenger’s lab at Duke University discovered that the plasma and endotracheal aspirate of COVID-19 ICU patients activated various TLRs, including TLR9. Aiming at identifying DNA-containing DAMPs as these agonists, they treated serum and ETA with DNA-scavenging MnO nanoparticles and observed strong reductions of the content of DNA, RNA and HMGB-1 and consequently, the activating effect on TLRs (14). Unfortunately, the hypothesised ‘DNA-containing DAMPs’ were not identified and serum, rather than individual DAMPs, was used in all TLR-induction tests. Although DNA/HMGB1 or DNA/histone complexes were likely contributing to the fraction of DNA-containing DAMPs, this study clearly shows that much more research will be required until certain inflammatory effects can be clearly related to specific DAMPs.

Several groups treated COVID-19 patients with recombinant human DNase I, administered as DNase I-coated nanospheres or as nebulized Dornase α, which is approved for the treatment of cystic fibrosis (117, 119, 122). I already mentioned the team from the University of Missouri (116). Subsequently, Hacer Kuzu Okur and colleagues at Acibadem Altunizade Hospital in Istambul (117) reported on 7^th^ of September 2020 that treatment of patients with Dornase α lead to significant clinical improvement in the radiological analysis, oxygen saturation and respiratory rate. These changes were associated with significantly decreased viral loads when comparing nasopharyngeal and oropharyngeal samples taken on the day before the treatment and after 7 days. In the same study, Dornase α decreased viral load and the negative effects of SARS-CoV-2 infection on cell proliferation in a realtime Vero cell culture system as well as *in vitro* NETosis by thawed adult human mononuclear cells (117). Three weeks later, Andrew G. Weber and coauthors from New York (118) reported on significant reductions in the production of proinflammatory cytokines by PBMCs and in the fraction of inspired oxygen in five mechanically ventilated COVID-19-patients who were treated with nebulized Dornase α and who were successfully extubated, discharged from hospital and remained alive (118). A further month later (on 20^th^ of October 2020), a multicentre team from S. Korea with first author Hee Ho Park (119) reported that treating plasma of COVID-19 patients with DNase-I significantly reduced the eDNA levels and NET formation. Moreover, DNase-I-coated nanospheres decreased eDNA concentrations in the blood, neutrophil activity, lung damage and mortality in a septic mouse model (119).

A team from Sweden with first author Jane Fisher (120) treated five severely ill COVID-19 patients with Dornase α and all of them became independent of mechanical ventilation and could be dismissed from the intensive care unit within 4 to 15 days of treatment. Immunofluorescence microscopy of sputum produced by these patients confirmed the infiltration by neutrophils and high abundances of NET-forming neutrophils in COVID-19 sputum, and ex-vivo treatment of the sputum from one patient demonstrated the rapid (within 10 min) degradation of NETs. In the same study, proteomic analyses of sputum allowed to identify both subtypes of immunoglobulin A and mucins, blood plasma proteins such as albumin, leukocyte proteins, and inflammatory/antiviral response proteins such as interferon-induced proteins as the most abundant proteins in COVID-19 and to reveal that the recovery due to Dornase α treatment was associated with a reduction in complement proteins, haemoglobin, lipopolysaccharide protein, and C-reactive protein, that is, proteins indicative of elevated innate immunity, cell damage and ongoing infection (120). In a study conducted by Andrew Toma and co-authors from Palm Beach Gardens Medical Centre (121), of 39 patients included in the study, 24 had reduced respiratory support requirements and out of 8 patients initially requiring mechanical ventilation, 7 were successfully extubated by the end of the study period.

A Greek team with first author Efstratios Gavriilidis (122) combined inhaled DNase with drugs aimed at controlling cytokine-mediated hyperinflammation: an inhibitor of the IL-6-receptor and a Janus Kinase (JAK)1/2 inhibitor. They report that the combined treatment was associated with significantly lower in-hospital mortality and intubation rate, shorter duration of hospitalization, and prolonged overall survival. As a notion of caution, in a clinical trial performed by the team at University of Missouri with 10 COVID-patients treated with Dornase α and 20 case controls and published 2021 with first author Zachary Holliday (188), the differences between treatment and control groups in static lung compliance and NET abundance in BALF were not maintained after terminating the treatment. The same study could detect on effect on NETs in the blood (188). In summary, a positive effect of Dornase α has so far only been shown for NETs on the surface, but not in the blood vessels of human COVID-19 patients.

Additional, although indirect support for the detrimental effects of DNA-driven inflammation is provided by the fascinating explanation as to why coronavirus infection does not cause disease symptoms in bats (189–193). The energy-demanding flight of bats unavoidably damages their mtDNA (191, 192). As an adaptation, bats have lost the inflammasome-forming PRRs AIM2 and IFI 16 and other IFN I-related genes, and they exhibit dampened transcriptional priming of several components of the NLRP3-inflammasome (192). Consequently, bats show almost no inflammatory immune response to cytoplasmatic or circulating self-DNA (191–193).

## 4 DAMPs and inflammasomes – a smart but dangerous liaison

“*DAMPs and TLRs have not received much attention in COVID-19*” Luis A. Silva-Lagos et al., 2021 (194)

### 4.1 DAMPs in COVID-19

Apoptosis represents a form of cell death of somatic cells long since known to be associated with abundant translocation of nuclear contents to cell surface blebs and ultimately, to the blood stream (195–197). Even more DAMPs are released when immune cells undergo inflammatory forms of cell death such as pyroptosis (22, 58, 181, 198, 199). The DAMP most frequently reported for COVID-19 is lactate dehydrogenase, a central enzyme of anaerobic glycolysis that is present in virtually all cells and that, therefore, reliably indicates cell death when occurring at high concentrations in the extracellular space (200–202). Other DAMPs which have been observed at elevated levels in the blood or plasma of patients with severe COVID-19 comprise high-mobility group box protein (HMGB)1 (51, 110, 111, 202–206), S100 proteins (108, 110, 202, 207–209), plasma hyaluronan (210), extracellular (e)ATP (211, 212), the antimicrobial peptide LL-37 (213, 214), histones (e.g (215–217), reviewed in (218, 219) and circulating self-DNA, including nDNA (13, 20, 21, 42, 46, 47), mtDNA (13, 18, 21, 42, 43, 48, 49), NET-associated cfDNA (201, 215, 220–222), histone-DNA complexes (219), and cfDNA of non-specified subcellular origin (45, 48, 50–54). As recently reviewed for sepsis (72), most of these studies reported a positive correlation of the plasma levels of at least some of the beforementioned DAMPs with the degree of disease severity (e.g., mild versus severe cases, COVID-19 patients at ICU admission versus healthy controls, ICU-admitted cases with fatal outcomes versus surviving patients, poor oxygenation status, patients with acute respiratory distress syndrome or with multisystem inflammatory syndrome in children, etc.).

As predicted by Polly Matzinger (62, 223), DAMPs trigger the activation of antigen-presenting cells, act as chemokines that recruit dendritic cells, macrophages, or T cells to the site of injury, or prime immune cells, i.e., they contribute to ‘trained immunity’ (58–60, 62, 224–226). The signalling function of lactate dehydrogenase remains a matter of discussion (227), but DAMPS *sensu strictu* such as HMGB1, eATP and cytoplasmatic and extracellular dsDNA prime the immune system for stronger responses to current and upcoming threats. One of the best studied examples of a DAMP-mediated immune priming is the activation of inflammasomes in two steps (59, 176, 177). Multiple lines of evidence support a critical role of inflammasomes in COVID-19 and – likely - its autoimmune complications (12, 13, 22, 29, 39, 181, 228–232) (for a review, see (39)). The lung and spleen tissue obtained from patients who died from COVID-19 exhibited higher densities of cells expressing NLRP3, IL-18, NF-κB and gasdermin D, and even HMGB-1, than age-matched controls who had died unexpectedly, but free of SARS-CoV-2-infection (13, 233). This observation indicates that the cells were already in the primed stage. Moreover, exosomes from patients with severe COVID-19 (but not light COVID-19 or healthy donors) induced the expression of NLRP3, pro-caspase-1 and pro-IL-1β in human endothelial cells, microvascular endothelial cells and liver endothelial cells (232). This last study appears to be the first empirical proof of concept showing that cell content released locally from infected cells can trigger systemic inflammatory effects in COVID-19. In addition, enhanced plasma levels of pyroptosis markers were detected in COVID-19 patients, and the levels of caspase -1 and IL-18 in serum correlated with the degree of COVID-19 severity (12, 22, 29, 220, 221), particularly in elderly patients (234). Neutrophils, macrophages and PBMCs from SARS-CoV-2-infected patients also exhibited active AIM2 or NLRP3 inflammasomes and enhanced expression of ASC-speck protein, caspase 1 or gasdermin-D (12, 13, 22, 29, 199, 221, 233). Correspondingly, specific inhibition of the NLRP3 inflammasome suppressed immune overactivation and alleviated COVID-19-like pathology in mice (235). Intriguingly, the SARS-CoV-2 N protein interacts directly with NLRP3 and promotes its binding to ASC, thereby facilitating the assembly of the NLRP3 inflammasome (236). Moreover, the E protein can form cation channels that allow for pyroptosis in the absence of active gasdermin-D (237).

### 4.2 Positive DAMPs/DNA-sensing feedback

“*Unless cell death is explicitly assayed in an experiment, the contribution of dying cells to the generation of DAMPs and alarmins may be missed*”. Melinda Magna and David S. Pisetsky, 2016 (61)

For many years, self-DNA was considered as immunologically inert, because self-DNA released from dying cells into the extracellular space was believed to be inaccessible to the intracellular DNA sensors. However, when bound to peptides or proteins, extracellular DNA can translocate into specialized compartments, including the endosome of pDCs, and then gain immunogenic properties (61, 72), e.g. via recognition by TLR9 (238–241). As pointed out earlier for the HIV-1-context (65) or sepsis (72), pyroptosis releases self-DNA together with cellular content, including HMGB and histones, i.e., two types of DAMPs which intrinsically possess DNA-binding properties. In fact, the nuclei of cells that die via pyroptosis usually remain intact and only show chromatin condensation, meaning that rather than as ‘naked’ DNA, genomic DNA is released as chromatin, hence, in an immunogenic form (177). Intratracheal instillation of mouse alveolar epithelia with the SARS-CoV-2 Spike protein and poly (I:C), a synthetic RNA mimic, triggered lung tissue injury and enhanced levels of circulating TNF-α and HMGB1 (203). The crucial role of HMGB1 in this effect is underlined by the observation that a treatment with anti-HMGB-1 antibody reduced these detrimental effects (203). Thus, a DAMP can increase the detrimental effects of virus-derived PAMPs, for which reason it seems likely that the positive association of diverse DAMPs with COVID-19 severity reflects a causal role, rather than being merely correlative. Also in NETs, self-DNA is bound to HMGB1 or histones, which prevents its degradation and facilitates its passage through membranes or its active uptake via endocytosis and/or receptor binding. In consequence, NETs are known to cause sterile inflammation or small vessel vasculitis, thereby generating microvascular damage that contributes to injury in the brain and lung tissues or favours certain autoimmune pathologies, including SLE (83, 242–244).

In addition, several host defence peptides (also termed ‘small secreted antimicrobial peptides’) possess DNA-binding properties (reviewed in (238)), and among these, LL-37 has been reported at increased levels in plasma of COVID-19 patients (213, 214). LL-­37 enhances the IL-­1β-­induced production of cytokines such as IL-6 in monocytes, likely via a DNA-independent mechanism (238), while extracellular histones induce the secretion of IL-1β, IL-6 and TNF-α from circulating monocytes (245). These cytokines, in turn, trigger a pro-inflammatory senescence-associated secretory phenotype in human umbilical vein endothelial cells, at least when they act in synergy (246). Thereby, cytokines that are released via pyroptosis can trigger the pyroptosis-independent release of cellular content from certain immune cells. Synergistic effects can also occur via the inflammasome-mediated axis: complexes formed by HMGB1 and dsDNA induce caspase-1 activation and IL-1β–release from monocytes at 25 times lower concentrations than each molecule alone, because they activate the AIM2 inflammasome (247).

A further, universal DAMP that is well known to act in synergy with other DAMPs or otherwise facilitates their perception is eATP. Extracellular ATP was found at elevated concentrations in the blood of patients with severe COVID-19 (211, 248). The increase in eATP concentrations could be related to two mechanisms: reduced activities of ectonucleotidases (211, 248) and opened pannexin channels (249). The eATP-mediated activation of P2X7 and the resulting production of intracellular reactive oxygen species (ROS) (250) can activate the NLRP3 inflammasome and the release of IL-1β, IL-12 and IFNγ from macrophages (251).

### 4.3 Anti-dsDNA autoantibodies in COVID-19

Anti-dsDNA autoantibodies represent a further class of proteins that enhance the accessibility of extracellular DNA to innate immune sensors. The release of cellular content during pyroptosis arguably means a massive liberation of potential autoantigens within an already pro-inflammatory environment. Therefore, multiple lines of evidence link inflammasome-mediated pyroptosis to the generation of anti-nuclear and/or anti-dsDNA antibodies. The detailed mechanisms that lead to the formation of these types of autoantibodies remain under discussion, although it seems reasonable to argue that the formation of antinuclear antibodies – a crucial pathogenic feature of SLE that has also been reported for patients following vaccination with BNT162b2 or mRNA-1273 (126) – is evidently facilitated by the fact that the nuclei of cells undergoing pyroptosis usually remain intact. More importantly in the context of the present considerations, DNA-containing immune complexes - like other DNA/protein complexes - present another means of facilitated uptake of cfDNA into immune cells, e.g., into monocytes (252), i.e., they allow for the sensing of self-DNA by TLR9 and thereby stimulate cytokine production (241, 252–254). In other words, pyroptosis releases self-DNA under conditions that are likely to render immunogenic properties to extracellular DNA, generating circulating self-DNA that is particularly prone to promote inflammation and autoimmunity (83, 244).

An enhanced prevalence of autoantibodies against nuclear self-antigens – including dsDNA - has been reported for patients with severe COVID-19 (86, 125, 255–258), in particular those patients who developed an autoimmune disease as a consequence of COVID-19 (259, 260). In addition, these autoantibodies were detected in the blood of patients who presented some of the adverse effects of the mRNA vaccines (261). For example, elevated levels of anti-nuclear and anti-dsDNA antibodies and, in one case, even anti-histone antibodies, occurred in various patients who developed autoimmune hepatitis after the first dose (132) or new onset SLE after the second or third dose of the BNT162b2 mRNA vaccine (129, 131, 132) or the mRNA-1273 vaccine (126).

### 4.4 RAGE shuttles extracellular DNA

At least one PRR is capable of sensing dsDNA outside of the cell: Although RAGE had been discovered as a receptor of advanced glycation end products, it functions as a multi-ligand PRR that senses diverse DAMPs, including HMGB1, S100 proteins and dsDNA, to activate NF-κB. RAGE is a transmembrane protein expressed in endothelial cells, pneumocytes, T and B cells, alveolar macrophages, monocytes and dendritic cells (262). In addition, proteolytic cleavage of the extracellular portion of membrane-bound RAGE can release soluble forms of RAGE, a process which is upregulated by inflammatory signals. Increased soluble RAGE in serum is also a predictor of mortality among COVID−19 patients (31, 32). The expression of RAGE was significantly increased in patients with severe COVID-19, along with its ligands, including S100 and HMGB-1 (263, 264), and a system-wide transcriptomic analysis identified RAGE among the strongly upregulated genes in the liver, and among the slightly, but significantly upregulated genes in the heart and lymph nodes of COVID-19 patients (Supplementary Table S2 to (265)). The causal involvement of RAGE in vascular injury and severe disease in COVID-19 patients was underlined by the reduced systemic inflammation and damage to blood vessels and increased survival of mice treated with pharmacological inhibitors of RAGE (262). Of particular interest in the context of the present work, RAGE can trigger inflammation by activating NF-κB, and it can sequester extracellular DNA to facilitate its transport to the endosome and thus, its exposure to TLR9 (30). Moreover, RAGE can trigger a prolonged activation of NF-κB that apparently overcomes several endogenous negative feedback control mechanisms (reviewed in (24)). Since RAGE itself is a NF-κB controlled gene, RAGE is particularly prone to contribute to potentially fatal feedforward scenarios, and NF-κB has been suggested to ‘universally enhance STING-mediated immune responses’ (266).

## 5 Spike and dsDNA sensors in the adverse events after COVID-19 vaccination

*Will scientists once again pursue “quick and easy solutions” in the hopes of stimulating a protective antibody response despite existing evidence that coronavirus vaccines (for animals) based on the S or Spike surface protein have largely been ineffective?*” Anne S. De Groot, 2003 (93)

### 5.1 Spike is expressed in vaccinees

The SARS-CoV-2 Spike protein, CoV-2 S, is the target encoded by the mRNA vaccines (267, 268). Although mRNA is normally assumed to have a short persistence time, it is highly likely that CoV-2 S is expressed on the cell surfaces of vaccinees over a considerable timespan (2, 17, 36, 182, 186, 269, 270). Vaccine mRNA has been detected at least 15 days after the first or second dose of BNT162b2 (271). Since only one (negative) sample covered a later time point (27 days) in this study, it seems likely that the persistence time of vaccine mRNA is longer than 15 days. In fact, another group detected vaccine mRNA in blood samples from 10 of 108 Hepatitis C Virus-infected patients at 28 days after vaccination (272), and in the germinal centres of axillary lymph nodes, vaccine mRNA was even detected at significant abundances at 37 days post vaccination and remained detectable at 60 days post vaccination (273). Similarly, spike itself was detected in the endothelial cells within inflamed areas of the brain and heart of a man who died three weeks after receiving his third vaccine dose with BNT162b2 mRNA (274), and in the plasma of individuals who exhibited postvaccination myocarditis, most of them within a week after vaccination with the BNT162b2 or the mRNA-1273 vaccine (134). In another study, circulating S1 was detected in the plasma of eleven out of 13 participants and, although it peaked on average 5 days after receiving the first dose of mRNA-1273, Spike was detected still after 15 days (275). Plasma S protein levels of 10 ng ml^-1^ were observed 10 days after vaccination in a woman with mRNA-1273-induced thrombocytopenia, while S1 concentrations reported for plasma of COVID-19 patients oscillate around 50 pg ml^-1^ and can reach maximum levels about 1 ng ml^-1^ (reviewed in (276)). Considering in addition that Spike expression in vaccinees is likely to occur in tissues and organs that in SARS-CoV-2-infected individuals are unlikely to be reached by circulating CoV-2 S, potential effects of Spike on host immunity and other vital functions must be taken seriously.

### 5.2 Syncytia formation and DNA damage have beneficial effects (for the virus)

In 2020, Jiang Hui and Mei Ya-Fang presented the hypothesis that CoV–2 S impairs DNA damage repair and thereby reduces the efficiency of an essential step in antibody production: the adaptive production of diverse antigen receptors via regulated dsDNA breaks and their subsequent repair (277). This work has been heavily criticised and finally was retracted. One of the critical voices was Derek Lowe (278) who, among other points, highlights that Jiang & Mei transfected DNA into specific and cultured cell lines, which do not necessarily respond like cells *in vivo.* Lowe reassures that - although “a lot of people are worried about Spike protein circulating around through the body”, “there is no evidence (and no particular reason to believe) that circulating Spike protein after vaccination, such as it is, gets taken up into other cell types and then taken into their nuclei” (278). Indeed, I am not aware of a report on CoV-2 S entering the nucleus, and cytosolic S protein does likely not trigger inflammation in epithelial cells (279). Still, I think that there is enough evidence that (and how) CoV-2 S can damage cells and – in particular – their DNA without entering the nucleus.

Evidence for a DNA-damaging effect of SARS-CoV-2 - and perhaps CoV-2 S - is accumulating (23, 36, 280–283). Comet assays and γ-H2AX immunostaining revealed elevated levels of DNA damage in SARS-CoV-2-infected Huh7 or Vero-6 (African green monkey kidney) cells (66, 190, 284, 285), but also in lymphocytes and cardiac tissue of deceased COVID-19 patients (286–288). In the human studies, DNA damage levels correlated with Il-6 expression in infected cells (285) and serum levels of IL-6 (287) and of other inflammatory ILs (286). Post-mortem transcriptomic analyses of cardiac tissues of COVID-19 patients revealed an enrichment of DNA damage and repair, heat shock, and cell cycle control among the predominant upregulated genes (288). An increase in oxidative stress and in the number of DSBs has been observed in PBMCs from older individuals at 24 h after vaccination with BNT162B2 (280), and several p53-controlled genes, including those related to apoptosis and DNA-repair, were overexpressed in PBMCs from a patient who developed myocarditis after BNT162b2 vaccination (hence, likely as a consequence of CoV-2 S expression in the absence of infection) (289). The consequences of these alterations comprise cell cycle arrest in the S1-phase, the activation of a pro-inflammatory senescence-associated secretory phenotype that exhibits elevated resistance to programmed cell death (190, 246), and enhanced expression of NF-κB and of the ACE2 promoter (290, 291).

In fact – as described earlier for HIV-1 (15, 292–294) - SARS-CoV-2 induces NF-κB, which inhibits the DNA damage-activated transcription factor p53. P53 is involved in cell-cycle control as part of the DDR, including the decision ‘DNA repair versus elimination of cells’ (295, 296). Correspondingly, p53-controlled genes were overexpressed in leukocytes from patients with severe COVID-19 (297). Moreover, SARS-CoV-2 degrades checkpoint kinase (CHK)1 (190), an effector downstream to Ataxia telangiectasia and Rad3 related protein (ATR) that earlier had been demonstrated to be induced in SARS-CoV-2-infected Vero-6 cells (284). Consequently, an inhibitor of ATR blocks the replication of SARS-CoV-2 after entering cells and thus, was identified as a potential anti-COVID drug that exhibits antiviral activity against SARS-CoV-2 in diverse cell types (298).

Zhou et al. (17) were among the first who discovered that the switch from a suppression to an (over-)induction of cytokine signalling associates with syncytia formation. Intriguingly, they also reported that CoV-2 S expression is sufficient to fuse cells. Several independent studies confirmed syncytia-formation by virus-free, Spike-expressing cells (23, 186, 269, 299–302). Syncytia formation, in turn, is inevitably associated with micronuclei formation, nuclear membrane blebbing and therefore, DNA damage, which ultimately leads to cell death (17, 23, 36). Later work confirmed that syncytia formation triggers IFN I signalling (23).

If we swich from the human perspective to the perspective of the virus, CoV-2 S-mediated detrimental effects of SARS-CoV-2 on host DNA become something that one simply would expect. Common knowledge holds that coronavirus Spike proteins control binding to the ACE2 receptor and subsequent fusion of the viral and the plasma - or endosomal membrane - of host cells, to facilitate viral entry (2, 186, 270) (**Figure 3**). However, viruses that replicate in the cytosol are short of raw material, i.e., nucleic acids, viruses gain a fitness advantage from supressing or escaping from the host immune system, and all viruses are under selective pressure to make the most efficient use of their small set of genes. Therefore, being a protein evolved to facilitate membrane fusion and the formation of pores, the Spike protein of SARS-CoV-2 has at least two additional jobs: it is employed to destabilise the nuclear envelope, and for syncytia formation (33, 34): a process associated with DNA damage, including the exposure of chromatin to cytoplasmatic DNA sensors (34, 36). For the latter, CoV-2 S possesses a signal that facilitates trafficking to the cell surface (269) and therefore is also expressed on the membrane of infected cells (182, 186, 269). Both effects benefit the virus: while nuclear membrane blebbing facilitates the liberation of the required ‘raw material’ from the nucleus, syncytia allow for viral spread without exposure to host antibodies, NETs or macrophages (**Figure 3**).

Evidently, more research will be required in this context. Most of the studies that reported syncytia-formation in virus-free systems that employed widely used model cell lines like HEK293T (human kidney cells), HeLa (human cancer cells), Calu-3 (human lung cells) or Huh-7 (human liver cells), which are overexpressing ACE2, and the used plasmid-based transfection systems further contribute to generate artificial conditions that limit a direct translation of the obtained results to realistic *in vivo* situations. On the other hand, the Spike protein encoded by the mRNA vaccines is stabilised in the prefusion-conformation to facilitate ACE2-binding and cell entry (303). It would be interesting to investigate to which degree syncytia formation is affected by this change versus ACE2 expression level (17, 23, 36, 269). In short, the virus uses CoV-2 S to damage it´s host´s DNA, and thereby enhances the lifetime of the infected cells and at the same time, upregulates ACE2 expression to decorate more cells with its entry receptor. By doing so, CoV-2 S activates certain elements of innate immunity which are also induced downstream to dsDNA sensing. Since CoV-2 S decorates more cells with the SARS-CoV-2-entry receptor, expression of this protein alone can be sufficient to trigger cell-to-cell fusion and subsequent nucleus-to cytosol shuttling of chromatin (17, 23).

Therefore, a scenario in which vaccination leads to an overstimulation of the innate immune responses downstream to dsDNA-sensors that generate the mentioned adverse effects appears definitively possible. In the following, I compile information on how CoV-2 S and SARS-CoV-2 infection affect elements of the dsDNA-sensing machinery and then develop a worse-case scenario describing hypothetical effects of Spike expression in an innate immune-environment that has already been primed by a prior SARS-CoV-2 infection. To base this scenario on – admittedly indirect – empirical evidence, I first highlight similarities between SARS-CoV-2 and HIV-1 that are related to HIV-1’s effects on DNA damage, dsDNA-sensing, inflammation, and autoimmunity.

### 5.3 Shared features: learning from HIV-1

“*Although there is much to learn about SARS, many lessons can already be drawn from our experience with HIV*”. Anne S. De Groot, 2003 (93)

Interfering with the DDR or directly damaging their host’s DNA, syncytia formation, and the induction of bystander cell death, as drivers of inflammatory responses that harm the host and benefit the virus: all these particularly important features are shared among HIV-1 and SARS-CoV-2 (304–306). Several inflammatory and autoimmune-related pathologies coincide among HIV-1-infected individuals, COVID-19 patients and vaccinated individuals who suffer from severe adverse effects. Based on the knowledge available to far, it seems very likely that these similarities result from the same mechanisms acting in all three contexts. Likewise, an enhanced prevalence of autoantibodies against nuclear self-antigens had already been reported for patients with HIV-1, particularly for HIV-1-infected patients presenting thrombocytopenia (91, 98, 307, 308). The present work is far from being the first one to highlight HIV-1 as an example from which a lot can (or could have been) learned for faster and more efficient response to the COVID-19 pandemic (93, 94, 309), and even the global phosphorylation landscape of SARS-CoV-2 infection revealed extensive similarities with the patterns in protein activation early during HIV-1 infection (310).

Since the early nineties of the last century, HIV-1 is known to damage the DNA of its host and activate genes involved in the DNA damage response (DDR) (294, 311–314), and HIV-1 is also well known for its ability to fuse its host cells and thereby reach 4-5 times higher reproduction rates (304, 315). In the case of HIV-I, the bipartite envelope glycoprotein (Env) performs the two essential functions of binding to receptors on the surface of target cells and fusion among host-cell and viral membranes, including the formation of a fusion pore to deliver the viral core into the cell cytoplasm (306). Apparently, the virus employs the same protein to destabilize the nuclear envelope. In this context, it seems important to recall that SARS-CoV and SARS-CoV-2 are closely related and that consequently, the S2 domain of the SARS-CoV spike protein and of CoV-2 S are highly similar (2, 34, 270). Importantly, SARS-CoV spike, in turn, shares multiple structural similarities with the gp41 unit of the envelope glycoprotein of HIV-1 (135, 316). Based on these similarities, which are underlined by the cross-reaction of CoV-2 S-directed non-neutralizing polyclonal antibodies with gp41 (317), these proteins can be expected to interact with cell membranes via the same mechanism.

Furthermore, while there should be no need to repeat that HIV-1 infects mobile immune cells (in particular CD4+ T helper cells), it seems worth to mention that evidence for SARS-CoV-2 doing the same is accumulating. SARS-CoV-2 has been shown to infect monocytes, macrophages and B-cells (22, 28, 318), and at least Pontelli et al (318) present evidence supporting a successful reproduction of the virus in these cells, although at low rates. Since excessive infiltration of mobile pro-inflammatory cells such as macrophages and T-helper 17 cells has been found in lung tissues of patients with COVID-19 (11), it appears at least possible that SARS-CoV-2 can apply a strategy that was considered unique to HIV-1: triggering local inflammation to attract mobile immune cells and infect these cells to achieve systemic distribution throughout the host.

A further shared element is the importance of eATP as a DAMP: HIV-1-infected target cells release ATP, which then acts on purinergic receptors to stimulate fusion between Env-expressing and CD4+ expressing membranes (319). eATP also favoured the infection of microglia with HIV-1, an effect that was associated with elevated levels of IL-6 and IL-18 and with changes in p53 activity (320). The relevance of this is underlined by the observation that an inhibitor of P2X receptors effectively inhibited cell-to-cell transfer of HIV-1 from productively infected CD4+ lymphocytes (321). Therefore, therapeutic applications of P2X7R antagonists seem promising tools to control infection with HIV-1 (322), but also SARS-CoV-2, but also SARS-CoV-2 (**Table 1**).

**Table 1:** Shared features of HIV-1, SARS-Co-2 and their envelope proteins.

	HIV-1	SARS-CoV-2	Env (gp41)	CoV-2 S	References	
					HIV-1	SARS-CoV-2
Entire virus						
Infection is associated with enhanced cf mtDNA or nDNA	**✔**	✔	n/a	n/a	(87–91)	(19, 20, 42–44, 47, 49, 52–56)
Triggers bystander cell death	✔	✔	✔	✔	(306, 323, 324)	(9, 13, 163, 325)
Opens pannexin channels to facilitate the release of eATP and cytokines	✔	✔		✔		(249)
						
Env/Spike-mediated functions						
Syncytia formation	✔	✔	✔	✔	(306, 323, 324)	(23, 182, 183, 269)
Destabilizes nuclear envelope	✔	✔	ns	✔	(306, 326)	(23, 190)
nDNA/chromatin release to cytosol	✔	✔		✔		(23, 190)
Mitochondrial membrane permeabilization		✔			(324)	
mtDNA release	✔	✔			(87, 327, 328)	
DNA damage	✔	✔		✔	(306, 324, 326)	(23, 183, 190, 280)
Induces ATM, ATR and/or p53 activation	✔	✔	✔	✔	(326)	(265, 297)
Features of the proteins						
Expressed on cell surface			✔	✔	(306, 329)	(182, 269)
Binds to cell surface receptors			✔	✔		
Controls fusion of virus and cell membrane			✔	✔	(323, 330)	(183)

n/a, not applicable; ns, not studied.

### 5.4 Effects of COVID-19 and CoV-2 S on innate immunity

Although viruses usually supress or avoid host immunity, CoV-2 S has been suggested to induce inflammation via a TLR2 (or TLR4) – and MyD88-dependent dependent activation of the NF-κB pathway in human and mouse macrophages (279), and also to trigger lung cancer progression, again via TLR2 (331). Likewise, intravenous administration of Spike or a stimulation of cultured cells with CoV-2 S induced the expression and release of TNF-α, IL-1β, IL-6 and IL-18 in PBMCs, macrophages, monocytes, lung epithelial cells, human umbilical vein cells as well as in lung, liver, kidney, and eye tissues (279, 332, 333). In fact, the S1 subunit alone was observed to induce an increased production of IL-6 and activation of NF-κB and in consequence, inflammation in endothelial cells (334), and circulating S1 induced the expression of TLR2, TLR4, NLRP3, IL-1β, TNFα and HMGB1 in rats at 24 h after treatment, and TLR2, TLR4, NLRP3 and IL-1β remained overexpressed even after seven days (335). Spike also has been reported to activate caspase 1 and the NLRP3 inflammasome in hematopoietic stem/progenitor cells and endothelial progenitor cells (336). Intriguingly, exposure to CoV-2 S of human umbilical vein cells activated NF-κB and ACE2 (333), while *vice-versa*, administration of an ACE2 inhibitor blocked the activation of inflammasome components by Spike (336). Quantification of lactate dehydrogenase (LDH) enzymatic activity in the culture medium confirmed pyroptotic cell death of these cells (336). Most of the beforementioned studies used commercially available, recombinant spike proteins produced in human cells (279, 333, 335), hamster cells (332), or *E. coli* (334), which opens the possibility of contaminations with immunogenic molecules that originate from the expression system. For example, bacterial lipopolysaccharides at very low concentrations as they have been detected as contaminations in commercial proteins activated human immune cells (337), and these endotoxins also signal via TLR4 (338). However, at least Sung et al. (339) used a pseudotyped lentivirus carrying the SARS-CoV-2 Spike protein, hence, a situation that resembles the vaccines. Assuming the reported *in vitro*-effects of CoV-2 resemble the situation *in vivo*, we can expect them to apply also to an infection with SARS-CoV-2. Indeed, nasopharyngeal epithelial cells of COVID-19 patients exhibited significantly higher expression of TLR2 and TLR4 as compared to controls (340), and TLR4 and its downstream elements (including Myd88, IRAK1 and TRAF6, and NF-κB - dependent genes) were significantly upregulated in PBMCs from 20 human COVID-19 patients (341).

### 5.5 Spike acting post-COVID-19: sketching the worse-case-scenario

The studies cited in 5.4. demonstrate that infection with SARS-CoV-2 S can prime the innate immune system for faster and stronger responses to any subsequent infection or other cell-damaging event. Indeed, plasma from COVID-19 patients exhibited increased in P2X7 expression (342). eATP can act as a second signal that activates inflammasomes, as shown by the observation that exposure to CoV-2 S followed by eATP triggered a stronger expression of pro-IL-1β, ASC, NLRP3 and gasdermin D in macrophages derived from COVID-19 patients than in SARS-CoV-2 naïve cells, and only patient-derived macrophages exhibited active ASC specks and increased secretion of TNF-α and IL-1β (229). Importantly, altered inflammasome and stress responses persisted after short-term patient recovery (29) and the differential responsiveness was maintained even by macrophages from fully convalescent COVID-19 patients after more than 50 days (i.e., after several cycles of monocyte renewal) (229). Likewise, TLR4 and its downstream elements (including Myd88, IRAK1 and TRAF6, and NF-κB - dependent genes) were significantly upregulated in PBMCs from 20 human COVID-19 patients (341).

These studies confirm that infection with SARS-CoV-2 causes a long-term reprogramming of the immune system, particularly in macrophages. Thus, it seems plausible that in vaccinees who had an infection before being vaccinated, the effects of SARS-CoV-2 expression (including DNA damage) occurred in the context of a primed DNA-sensing machinery: a situation that can strongly enhance its immunogenic potential. Others discovered that S1 and S2 proteins administered intraperitoneally triggered enhanced concentrations of IL-6, IL-1β, and TNFα (16 hr post treatment) in WT mice but not in mice lacking TLR2, which indicates a role of TLR2, rather than TLR4 (279). In addition, CoV-2 S induced an enhanced release of ATP and IL-1β from human lung epithelial cells (249) and cultured microglial cells (BV2 line) and in the latter, it also induced the expression of the purinergic eATP receptor P2X7 (343).

I conclude that SARS-CoV-2-mediated immune priming can enhance the DNA-damaging and pro-inflammatory effects of CoV-2 S and cause certain responses to pass a threshold or point of no return, reaching those dimensions that we see in the severe adverse effects of the Spike-based mRNA vaccines. Even phase III vaccine trials usually exclude individuals who show preexisting immunity due to previous infection, but the vaccination campaigns included significant proportions of the entire population at a time point at which many people had already passed through an infection with SARS-CoV-2, and pre-existing immunity was seldom checked in these mass vaccination events. In summary, immune priming represents an example of a mechanism that could generate different outcomes of vaccination depending on an earlier – perhaps non-symptomatic and not detected – infection with SARS-CoV-2.

## 6 Lessons and recommendations

Altogether, these findings motivate several recommendations.

### 6.1 Consider the ‘old’ literature and knowledge from other pathologies

Most of the published work on the SARS-CoV S protein and the similarities of SARS-CoV-2 with HIV-1 was ignored in the searches for new or pre-existing drugs to treat COVID-19 patients, and in the scientific activities involved in vaccine development. Perhaps as a consequence, on the one hand, patients were treated with IFN and indeed, slightly (although not significantly) more people died in the treatment group (344). On the other hand, DNases or DNA scavengers were hardly considered: to the best of my knowledge, all studies in which patients were treated with Dornase α are cited in chapter 3.5 (116–119, 121, 188, 222), and the obtained results – in particular the survival of all involved patients - clearly demonstrate the beneficial effects of anti-DNA treatments. As suggested by Anne de Groot and an international group with lead author Nevan Krogan (93, 310), existing knowledge should be considered even when it must be found in 20 years ‘old’ papers. Moreover, searches for similar pathogens should define ‘similarity’ with a focus on shared strategies or functions of the pathogen that generate similar effects on the interactions with the host, rather than on ‘taxonomic similarity’ defined at the level of sequences.

### 6.2 Don’t forget about the self-DNA!

“*We did not expect an RNA virus like SARS-CoV-2 to be sensed by the DNA sensor AIM2*”. Caroline Junqueira et al., 2021 (345)

In addition to the beforementioned issues, it seems that the dominance of the Janeway paradigm (346, 347) has significantly hindered a full appreciation of the immunogenic and pro-inflammatory effects of self-DNA. In fact, the author is aware of a few concrete cases in which the publication of results supporting the immunogenic effects of self-DNA or the danger model in general was significantly slowed down (personal communications by Polly Matzinger and Verena Kopfnagel, and own observations (348)). As a consequence, it seems that cfDNA, and nDNA in particular, remains strongly under-investigated. Even among those studies that originally linked the inflammation-related complications in COVID-19 to dsDNA sensors, only a single one has directly quantified mtDNA and nDNA and none has treated cells with natural DNA, as done, for example, in some of the studies that investigated the role of cfDNA in trauma (69). Nevertheless, even among the classical studies on trauma, three studies focused on mtDNA (66, 67, 170), although the fourth study found that serum IL-6 levels, inflammation and critical illness correlated with the levels of nDNA, not mtDNA (68). Likewise, most of the clinical studies that quantified plasma DNA concentrations as a possible marker of severe COVID-19 have quantified only mtDNA, without citing any empirical evidence for the assumed non-activity of nDNA.

Evidently, future work will have to provide definitive evidence for a causal role of cfDNA in severe COVID-19 or the vaccine-triggered adverse events. Still, beyond doubt, cfDNA levels in the blood or plasma are a useful biomarker of disease severity in COVID-19 patients and likely, a predictor of certain adverse effects of the vaccines. Although it would be difficult to follow the advice of The European Academy of Allergy and Clinical Immunology (EAACI) that “all clinical and laboratory information should be collected and reported…to understand the specific nature of the reported severe allergic reactions” (349), it seems mandatory to include cfDNA of both mitochondrial and nuclear origin and anti-dsDNA antibodies in the list of standard laboratory information that should be obtained for patients that suffer from autoimmune-related or chronic inflammatory pathologies and for all participants in future vaccination trials.

### 6.3 Consider synergies and other mechanisms that create context dependency

“…*studies … are often performed using a single well-defined ligand. However, …cells usually receive multiple inputs or experience many environmental alterations simultaneously*.” Andrea Ablasser, 2019 (123)

Multiple reports on stronger responses to a certain trigger shown by cells from COVID-19 patients or in response to DNA bound to other DAMPs provide examples of clinically important synergistic effects. In particular, extracellular self-DNA gains immunogenic properties when bound to other DAMPs or extracellular vesicles, independently of the concrete pathosystem. Therefore, the suggested effects can occur in basically all situations that comprise tissue damage, particularly when this damage occurs in the context of pre-activated innate immune signalling. The antibody-assisted infection of cells and the priming of certain immune responses by viral proteins represent further examples of synergies that can create a significant level of context dependency in the obtained results. As outlined by Andrea Ablasser, such effects are likely to be overlooked in experiments that expose naïve cell lines under controlled conditions to a single ligand (123). If we, e.g., study human defensin 5 (HD5) and the human cathelicidin known as LL-37 each in an isolated manner, we will observe that LL-37 binds to the carboxypeptidase domain of human ACE2 even stronger than HD5. From this observation, we could conclude that LL-37 bears a great potential to be tested as an anti-SARS-CoV-2 peptide, because it blocks the entry receptor of the virus (350). However, *in vivo*, the release of LL-37 and human β-defensin 3 might take place in the presence of pyroptotic cells or other sources of cfDNA. In case that – as shown earlier – these proteins facilitate the uptake of self cfDNA into pDCs and monocytes (239) and thereby potentiate its immunogenic potential to trigger pro-inflammatory effects through the TLR9 pathway (351), the net outcome of treating severe COVID-19 patients at the later stages of the disease with LL-37 might be fatal, as SARS-CoV-2 infection has significantly declined, while the inflammatory response escalates dramatically, becoming predominant (67).

## 7 Drawbacks and limitations of the present work

8.1 First, and most importantly, the author of this contribution has no medical degree but rather, is a plant ecologist who discovered the immunogenic effects of self-DNA in plants. I can only hope that the readers appreciate the non-specialist’s perspective to an important medical topic and forgive me all the technical errors.

8.2 Second, although it seems a hackneyed wisdom, correlations do not necessarily mean causality, and similar or even identical symptoms do not necessarily result from the same mechanism.

8.3. Final evidence for a causal role of elevated levels of cytosolic or extracellular self-DNA as a driver of systemic inflammation in severe COVID-19 remains to be provided, and it will be difficult to separate the TLR-mediated pro-inflammatory effects of CoV-2 S from pro-inflammatory effects that are – as suggested by the present work – caused by self-DNA release due to CoV-2 S-mediated damage to genomic DNA and the nuclear envelope. However, the results obtained by Park & col with their mouse model (119) and Oku & col (117) and Weber & col (118) with human COVID-19 patients provide very strong evidence in favour of this role.

8.4 Third, most of the discussed adverse effects are not exclusive for the mRNA-based COVID-19 vaccines but have also been observed in patients that received adenovirus-based COVID-19 or non-COVID-19 vaccines, although at much lower frequency. In addition, all these effects were also present in COVID-19 patients, and most were more frequent among COVID-19 patients as compared to vaccinees. Even autoantibodies are also being reported from COVID-19 patients (143, 256, 258). The other way round, several of the studies that tested for plasma concentrations of nDNA or mtDNA failed to detect a statistically significant difference between light and severe cases or between deceased and discharged patients (21). Likewise, I am only aware of a single report that connects adverse effects of the COVID-19 mRNA vaccine to inflammasome-activation (352).

8.5 If all the above was true, how do we explain that the vast majority of vaccinees did not present adverse events, at least no severe ones? And how can we explain contradictory reports, e.g. that “chronic stimulation with SARS-CoV-2 Spike protein does not trigger autoimmunity” (353).

8.6 As this paper focuses on DAMP/DNA-dependent mechanisms shared by HIV-1 and SARS-CoV-2, various further, alternative (but non-exclusive) explanations, including effects of the longer activity of the mRNA or direct effects of blocking the ACE2 receptor, have not been ruled out. In addition, I have focused on the mechanisms in order to discuss all interpretations of pro-inflammatory and immunogenic effects of DNA, because detrimental innate immune activation and inflammation represent the shared element between HIV-1, COVID-19 and adverse vaccine effects. For example alternative interpretations leading to different conclusions exist even for the observation of elevated levels of cfDNA in vaccinee plasma, which has been interpreted as a favourable indicator of the formation of memory B cells after vaccination (127). In general, we must not forget that DNA-induced inflammation and pyroptotic cell death usually represent adaptive immune responses which benefit the host.

## 8 Conclusions and outlook

Several lines of - mainly correlative - evidence suggest that extracellular self-DNA acting as a pro-inflammatory DAMP represents a shared element that contributes to diverse life-threatening complications in patients infected with HIV-1 and with SARS-CoV 2, and that a contribution to some of the severe adverse events after vaccination with the mRNA vaccines represents a possibility that merits further investigation. Evidently, any attempt to explain the outcomes of complex, systemic processes with a single factor is determined to fail, and the present work by no means tries to claim that immunogenic DNA is the only important factor. However, self-DNA and other DAMPs have the specific features to prime numerous elements of the innate immune response and to engage in positive feedforward mechanisms and synergistic effects, including the formation of closed loops that lead to self-induction phenomena. For example, extracellular DNA at normally non-immunogenic concentrations can gain immunogenic properties when binding to HMGB1, then activate TLR9 which ultimately leads to pyroptosis and the release of more DNA and HMGB1. Context-dependent processes that eventually enter feed-forward dynamics are notoriously difficult to monitor, and more so in unbiased screenings. Therefore, immunogenic self-DNA represents a prime candidate of a frequently overlooked important factor whose true role hardly becomes evident in the classical one-treatment experimental designs. In the light of this possibility, the general tendency to tone down the adverse effects of SARS-CoV-2 vaccines as “often troubling but may merely reflect transient production of type I interferons, a normal physiological response to contact with invading microorganisms” (354) appears in a different light.

Under certain conditions, usually transient effects might pass a certain threshold and then become subject to very different dynamics. Therefore, all those of the above-described mechanisms that can form positive feedback loops can potentially lead fatal outcomes under certain circumstances. Whether SARS-CoV-2 Spike causes DNA damage or inhibits the DDR remains to be shown. However, the consequences of DNA damage- even at low levels - on IFN I signalling and inflammation could be potentiated if concurrent damage to the nuclear envelope facilitates DNA release from the nucleus and subsequent sensing by of cytosolic DNA or when damage to the cell membrane facilitates the release of DNA together with other DAMPs and thus, the formation of immune complexes.

At the very least, our question from 2019 “To what degree can … the use of DNA scavengers developed as specific treatments for cancer or diverse autoimmune diseases be adapted to treat individuals with chronic HIV-1 infection?” (65) has now been answered for SARS-CoV-2-infected patients, although only few patients received this kind of treatment and its transferability to intravascular NETs or other DNA-dependent inflammatory pathologies remains to be investigated (117, 118). It would be encouraging if future research and vaccine development efforts would reflect an increased awareness of the potential detrimental effects of immunogenic self-DNA and of the existence of treatment options which – as stated by Okur & col (117) for Dornase α – “are being administered to human patents since decades”, although in a different pathological context.

## Data availability statement

The original contributions presented in the study are included in the article/supplementary material. Further inquiries can be directed to the corresponding author.

## Author contributions

MH: Conceptualization, Writing – original draft, Writing – review & editing.
